# Effect of National Schistosomiasis Control Programme on *Taenia solium* taeniosis and porcine cysticercosis in rural communities of Tanzania

**DOI:** 10.1016/j.parepi.2016.08.004

**Published:** 2016-08-28

**Authors:** Uffe Christian Braae, Pascal Magnussen, Wendy Harrison, Benedict Ndawi, Faustin Lekule, Maria Vang Johansen

**Affiliations:** a Section for Parasitology and Aquatic Diseases, Department of Veterinary Disease Biology, Faculty of Health and Medical Sciences, University of Copenhagen, DK-1870 Frederiksberg, Denmark; b Centre for Medical Parasitology, Faculty of Health of Medical Sciences, University of Copenhagen, DK-1353 Copenhagen, Denmark; c Faculty of Medicine, School of Public Health, Imperial College London, United Kingdom; d Bora Professional Consultancy Services, Iringa, Tanzania; e Faculty of Agriculture, Sokoine University of Agriculture, Morogoro, Tanzania

**Keywords:** *Taenia solium*, Taeniosis, Mass drug administration (MDA), Cysticercosis, Preventive chemotherapy

## Abstract

*Taenia solium* is found throughout sub-Saharan Africa and co-endemic with schistosomiasis in many regions. *Taenia solium* leads to taeniosis and neurocysticercosis - the leading cause of preventable epilepsy globally. This study aimed to assess the effects of the National Schistosomiasis Control Programme on prevalence of taeniosis and porcine cysticercosis over a four year period in Tanzania. School-based mass drug administration (MDA) of praziquantel was carried out based on schistosomiasis endemicity. Four human and five porcine cross-sectional surveys were carried out from 2012 to 2015 in Mbozi and Mbeya district in Tanzania. Three rounds of school-based MDA of praziquantel were delivered in Mbozi and two in Mbeya. The prevalence of taeniosis and porcine cysticercosis was estimated annually. Stool samples were collected from humans and prevalence of taeniosis estimated by copro-Ag-ELISA. Blood samples from pigs were collected to estimate cysticercosis prevalence by Ag-ELISA. “Track-and-treat” of taeniosis cases was carried out after each survey. In total 12082 stool samples and 4579 porcine serum samples were collected. Significantly fewer children (≤ 15) from Mbozi were infected throughout the study than children from Mbeya who showed a significant decrease in copro-Ag prevalence after the first treatment only. During the final survey in Mbozi the prevalence of taeniosis in adults (1.8%) was significantly lower (p = 0.031, OR 0.40, CI: 0.17–0.89), compared to baseline (4.1%). The prevalence of porcine cysticercosis (8%) had also dropped significantly (p = 0.002, OR 0.49, CI: 0.32–0.76) in this district compared to baseline (13%), whereas no significant difference was seen in Mbeya compared to baseline. The study suggests that three rounds of MDA targeting schistosomiasis in school-aged children combined with ‘track-and-treat’ contributed to a reduction in prevalence of *T. solium* in this population, and also had a spillover effect on adults in treated areas as well as reducing the prevalence of *T. solium* in the intermediate pig host population. Elimination of *T. solium* in this area would require a One Health approach.

## 1 Introduction

The zoonotic tapeworm *Taenia solium* is prevalent throughout sub-Saharan Africa (Braae et al., 2015c), and constitutes a serious, but preventable, agricultural and public health problem. In 1993 *T. solium* taeniosis/cysticercosis was declared eradicable by the International Task Force on Disease Eradication (ITFDE), but to date no large-scale control programmes have been implemented in sub-Saharan Africa. A larger scale initiative to eliminate *T. solium* has recently been trialled in Peru (Garcia et al., 2016). Due to the zoonotic properties of *T. solium,* a cross-disciplinary One Health approach involving both the agricultural sector and the human health sector, targeting both human and porcine hosts is likely to be essential to eliminate the parasite. Models have shown prevalence to revert shortly after intervention targeting only one host (Kyvsgaard et al., 2007).

Schistosomiasis is found throughout sub-Saharan Africa and co-endemic in many areas with *T. solium* taeniosis/cysticercosis (Braae et al., 2015c). National scale control programmes targeting schistosomiasis have been implemented in over 30 African countries. In Tanzania the National Schistosomiasis Control Programme (NSCP) carries out school based MDA with praziquantel at 40 mg/kg. The frequency of administration is dependent on the prevalence of schistosomiasis, in accordance with WHO guidance. Praziquantel is also considered efficacious against taeniosis at a dose of 5–10 mg/kg (Pawlowski, 1991). Therefor there is potential for MDA using praziquantel on both helminth species.

This study aimed to investigate the impact of a school based NSCP on taeniosis and porcine cysticercosis by assessing the effect of repeated rounds of praziquantel MDA at 40 mg/kg in combination with treatment of taeniosis cases identified during the study, in two areas co-endemic for *T. solium* taeniosis/cysticercosis and schistosomiasis.

## 2 Materials and methods

### 2.1 Study design

**Fig. 1:**
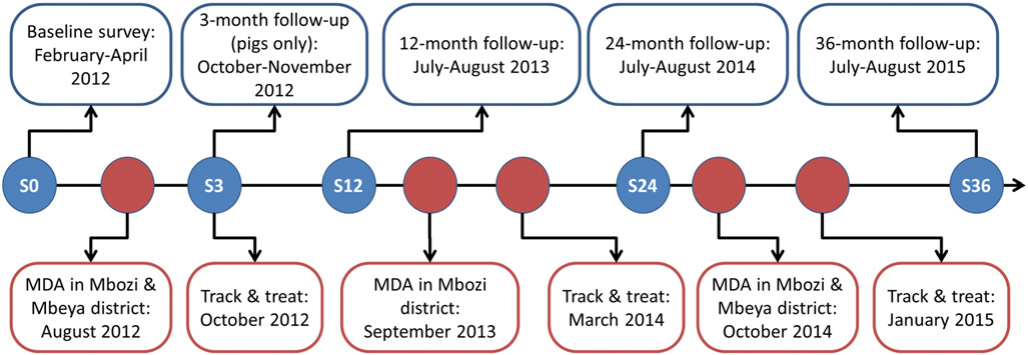
Study design covering surveys (S) at month-0 (S0) to month-36 follow-up (S36) from 2012 to 2015 in Mbeya and Mbozi district, Tanzania. “Track-and-treat” was attempted on all taeniosis cases identified during the study period.

The study consisted of multiple cross-sectional surveys carried out in the two districts Mbeya and Mbozi in Tanzania from 2012 to 2015 (Fig. 1). MDA of praziquantel at 40 mg/kg to school-aged children were carried out three times in Mbozi district and twice in Mbeya district by the NSCP.

**Fig. 2:**
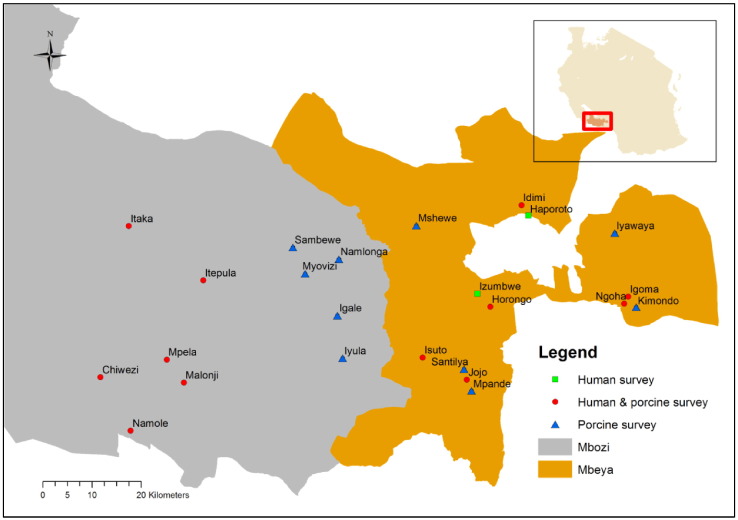
Map of study area, Mbeya and Mbozi district in Tanzania, showing where human stool and porcine blood samples were collected in the period 2012 to 2015.

Stool samples were collected in 14 villages from the human population and has been described elsewhere (Braae et al., 2015b), except for an additional survey preformed in July/August 2015 using identical methodology. Serum samples were collected from pigs in 22 villages and has also been described elsewhere (Braae et al., 2014), with the exception of the last two surveys carried out in July/August 2014 and July/August 2015 using identical methodology (Fig. 2).

### 2.2 Data collection and ELISA analyses

During each survey approximately 1500 humans and 400 pigs were targeted in each district. Human stool samples were analysed for taeniosis using the copro-Ag-ELISA (Allan et al., 1990, Mwape et al., 2012). Porcine serum samples were analysed for cysticercosis using the B158/B60 Ag-ELISA (Brandt et al., 1992, Sikasunge et al., 2007).

### 2.3 Ethical considerations

The National Institute for Medical Research (NIMR) in Tanzania approved the study, reference number NIMR/HQ/R.8a/Vol. IX/1216 as did the Imperial College Research Ethics Committee (ICREC), reference no. ICREC_11_3_6. Permission to conduct the study was also sought through Sokoine University of Agriculture in Morogoro, Tanzania, in addition to regional, district, and local village authorities. All treatments were carried out and overseen by the NTD secretariat of the Ministry of Health, Community Development, Gender, Elderly and Children. Prior to each survey involving humans, study villages were visited and the communities informed about the upcoming survey. Members of the communities were given the opportunity to ask questions and seek more information about the study. Written informed consent was obtained from all participants in the human part of the study after they were informed about the aim, risks, and benefits of the study. If the person was under 18, consent was sought from a parent or guardian following assent from the child. After each survey, and upon receiving the laboratory results of the copro-Ag-ELISA, villages in the study area were visited, and village leaders, school headmasters, and head teachers were informed about the results of the survey. Copro-Ag-ELISA positive individuals were offered anthelminthic treatment, either a single dose of 2 g niclosamide for adults and 50 mg/kg for children or praziquantel at 10 mg/kg, within 5–8 months of sample collection. Forms to report side-effects of the MDA were distributed to schools in the study villages and all individuals treated during the ‘track and treat’ were provided with a contact number to a medical doctor in case of side-effects. All animals were handled in strict accordance with good animal practice as defined by the OIE's Terrestrial Animal Health Code for the use of animals in research and education. Oral consent for porcine blood collection was sought from the farmer after information about the aim, risks, and benefits of the study were provided. Pigs found positive for *T. solium* cysticercosis, were treated at the subsequent visit with oxfendazole (30 mg/kg) using an oral drench gun with synanthic 9.06 suspension, Merial, France, (batch no. F10701B). Owners of treated pigs were instructed both orally and in writing that the pork was unfit for human consumption if the pig was slaughtered within 28 days of treatment with oxfendazole.

### 2.4 Statistical methods

Human survey data were entered into EPI info 7 (http://wwwn.cdc.gov/epiinfo/7/) and transferred to an Excel spread sheet (Microsoft Office Excel 2010®). Porcine survey data was entered directly into an Excel spread sheet (Microsoft Office Excel 2010®). All statistical analyses were performed in R (R Core Team 2015). Variables in the logistical regression were considered significant if the p-value was < 0.05. Odds ratios (OR) and 95% confidence intervals (CI) are provided for significant variables. All analyses were performed using the full dataset from 2012 to 2015 on both porcine and human surveys (Braae et al., 2014, Braae et al., 2015b).

### 2.5 Role of the funding source

The funder of the study had no role in design of the study, no access to the data, and did not participate in the analyses, interpretation, writing, or decision to submit the manuscript. The corresponding author had access to all the data and is responsible for the decision to submit the manuscript for publication.

## 3 Results

### 3.1 Taeniosis survey

In total 3018 stool samples from S36 were analysed for copro-antigens in the two districts (Table 1). During the whole study period from 2012 to 2015, 12082 stool samples were collected and analysed for taeniosis. Based on logistic regressions with baseline data (S0) from Braae et al. (2015b) as reference and controlling for sex and age, children (≤ 15 years) in Mbozi, the area that had received MDA three times, had significantly decreased odds of being infected at S12 (p = 0.005, OR 0.12, CI: 0.02–0.42), S24 (p = 0.001, OR 0.24, CI: 0.10–0.55), and S36 (p = 0.002, OR 0.04, CI: 0.01–0.21), and a drop in prevalence from 2.3% (Braae et al., 2015b) to 0.1%. The identical analysis for children (≤ 15 years) from Mbeya, the area that had only received treatment twice, showed only a significant decrease from baseline (S0) after the first round of MDA at S12 (p = 0.036, OR 0.20, CI: 0.03–0.74). Among the children in Mbeya, decreasing age was associated with increased odds of taeniosis infection (p < 0.001, OR 0.70, CI: 0.59–0.82). Adults in Mbozi had gradual decreasing odds of infection at each subsequent time point, but odds were only significantly different from baseline after three rounds of MDA at the last time point (S36) (p = 0.031, OR 0.40, CI: 0.17–0.89), where prevalence had dropped from 4.1% (Braae et al., 2015b) to 1.8%. In the adult population increasing age (p = 0.026, OR 1.02, CI: 1.00–1.04) and being male (p = 0.028, OR 1.83, CI: 1.08–3.18) was significantly associated with infection in Mbozi. No significant difference was seen among the adults in Mbeya in any of the surveys, but age was associated with infection (p = 0.034, OR 1.03, CI: 1.00–1.06).

**Table 1:** Study population characteristics and prevalence of taeniosis in the two districts Mbozi and Mbeya during the last 36-month follow-up survey in July/August 2015 (S36).

Survey	S36	
District	Mbozi	Mbeya
Stool samples	1517^a^	1501^b^
Males %	46	43
Females %	54	57
Children (≤ 15 years)	1070	1076
Adults (> 15 years)	444	425
Taeniosis (%)	9 (0.6)	7 (0.5)
Taeniosis in children (%)	1 (0.1)	4 (0.4)
Taeniosis in adults (%)	8 (1.8)	3 (0.7)

a Age was not recorded for 3 individuals.

b Sex was not recorded for 1 individual.

**Fig. 3:**
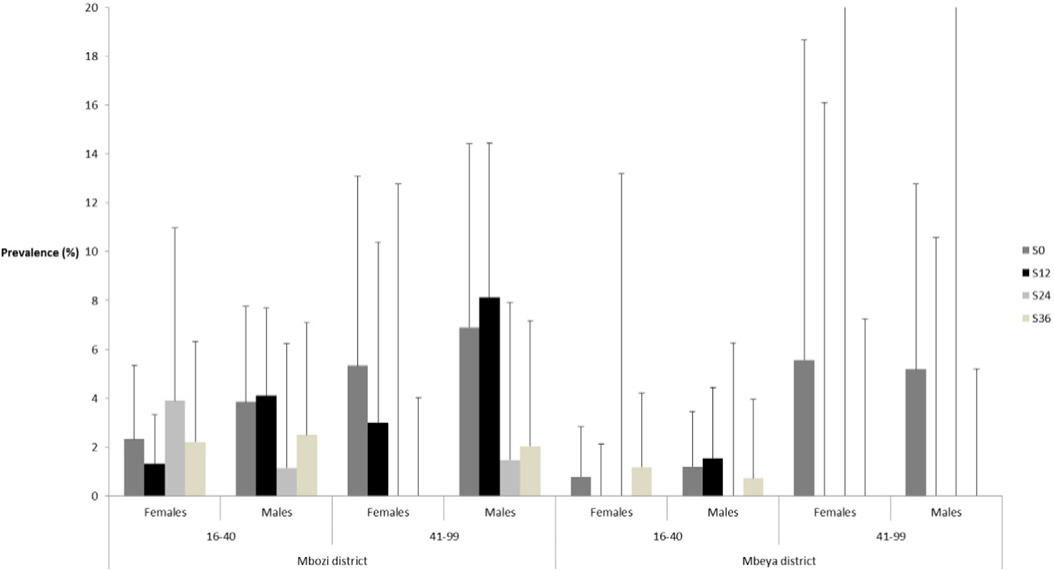
Taeniosis prevalence among adult age groups in areas receiving school based MDA in Mbeya and Mbozi district, Tanzania at Feb-Apr 2012 (S0), Jul-Aug 2013 (S12), Jul-Aug 2014 (S24), and Jul-Aug 2015 (S36).

Approximately five months after the 24 month time point (S24) all positive taeniosis cases, 12 in Mbozi and eight in Mbeya, were “tracked-and-treated” with praziquantel at 10 mg/kg. Five months after the final survey (S36) 15 out of the 16 positive individuals were treated during “track-and-treat” with 2 g niclosamide for adults and 50 mg/kg for children. It was not possible to quantify the effect of the “track-and-treat” strategy. Although sample sizes were relative small the results indicate that taeniosis is prevalent among older individuals (Fig. 3) which was also supported by the logistical regression analysis. No side-effects were reported during the study period from any of the schools or individuals treated during the study.

### 3.2 Porcine cysticercosis surveys

In each district > 400 pigs were sampled during each of the five surveys (Table 2). During the whole study period from 2012 to 2015, 4579 porcine blood samples were collected and analysed for porcine cysticercosis. The prevalence of porcine cysticercosis increased in both districts significantly after the first three months. Potential causes of this increase in prevalence have been presented elsewhere (Braae et al., 2014). There was no difference in the proportion of pigs being kept confined during the study period within each district, but significantly more pigs were kept confined in Mbeya compared to Mbozi at each time point (Table 2).

**Table 2:** Porcine study population characteristics, production system practised, and prevalence of porcine cysticercosis, in the two districts Mbozi and Mbeya from five cross-sectional surveys during 2012 to 2015.

Survey	S0^a^ Mar/Apr 2012		S3^a^ Oct/Nov 2012		S12^a^ Jul/Aug 2013		S24 Jul/Aug 2014		S36 Jul/Aug 2015	
District	Mbeya	Mbozi	Mbeya	Mbozi	Mbeya	Mbozi	Mbeya	Mbozi	Mbeya	Mbozi
Pigs	408	414	406	406	528	470	474	486	450	537
Sex										
Male %	39	43	38	36	39	34	41	39	42	39
Female %	61	57	62	64	61	66	59	61	58	61
Mean age in months (± SD)	7.3 (4.3)	7.5 (4.8)	9.2 (6.8)	8.6 (4.9)	9.8 (6.9)	9.0 (4.8)	9.2 (6.1)	9.6 (6.3)	7.7 (4.5)	8.8 (5.7)
Production system										
Confined %	93.3	62.0	92.4	56.4	89.0	59.5	90.9	57.3	95.1	64.0
Semi-confined %	4.2	21.0	3.2	9.1	5.3	27.8	5.1	29.4	4.0	22.8
Free-range %	2.5	17.0	4.4	34.5	5.7	12.6	4.0	13.3	0.9	13.2
Sero-prevalence %	18	13	27	21	21	19	15	12	14	8

a Data have been presented previously on a regional level (Braae et al., 2014).

**Fig. 4:**
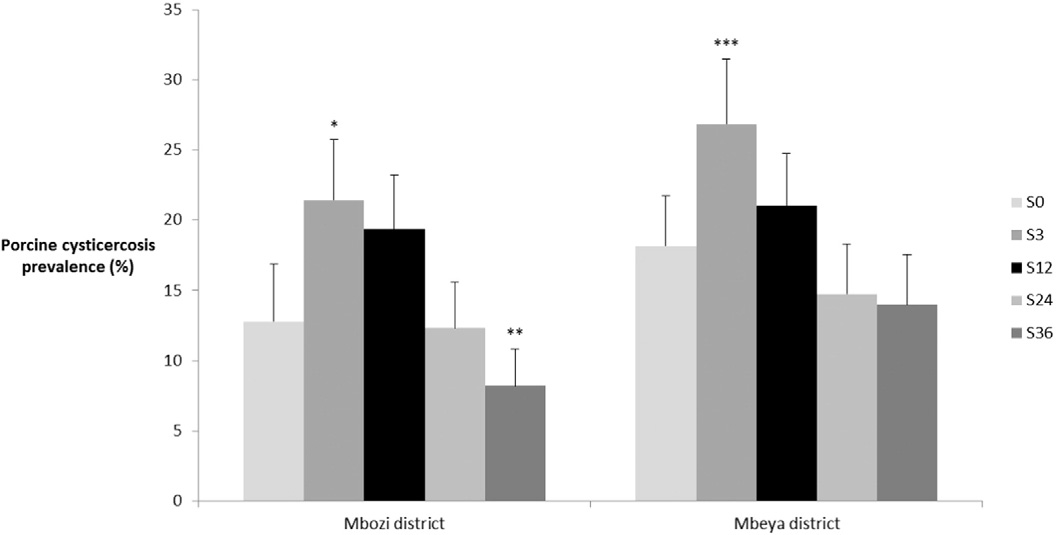
Porcine cysticercosis prevalence in the two districts Mbozi and Mbeya, Tanzania at Feb-Apr 2012 (S0), Oct-Nov (S3), Jul-Aug 2013 (S12), Jul-Aug 2014 (S24), and Jul-Aug 2015 (S36). Asterisk (*) marks significant difference with baseline (S0) based on logistical regression with *p = 0.01–0.05, **p = 0.001–0.009, and ***p < 0.0001. Error bars depict the 95% binomial confidence intervals based on number of positives and sample size.

Compared to the baseline (Fig. 4), a significant decrease in prevalence (13% to 8%) of porcine cysticercosis (p = 0.002, OR 0.49, CI: 0.32–0.76) was reported in Mbozi at the 36-month time point (S36) after three rounds of MDA. For Mbozi district, also the age of the pigs were found to be a significant risk factor of disease (p < 0.001, OR 1.04, CI: 1.02–1.06). No risk factors were identified for Mbeya district.

### 3.3 Taeniosis and porcine cysticercosis

**Fig. 5:**
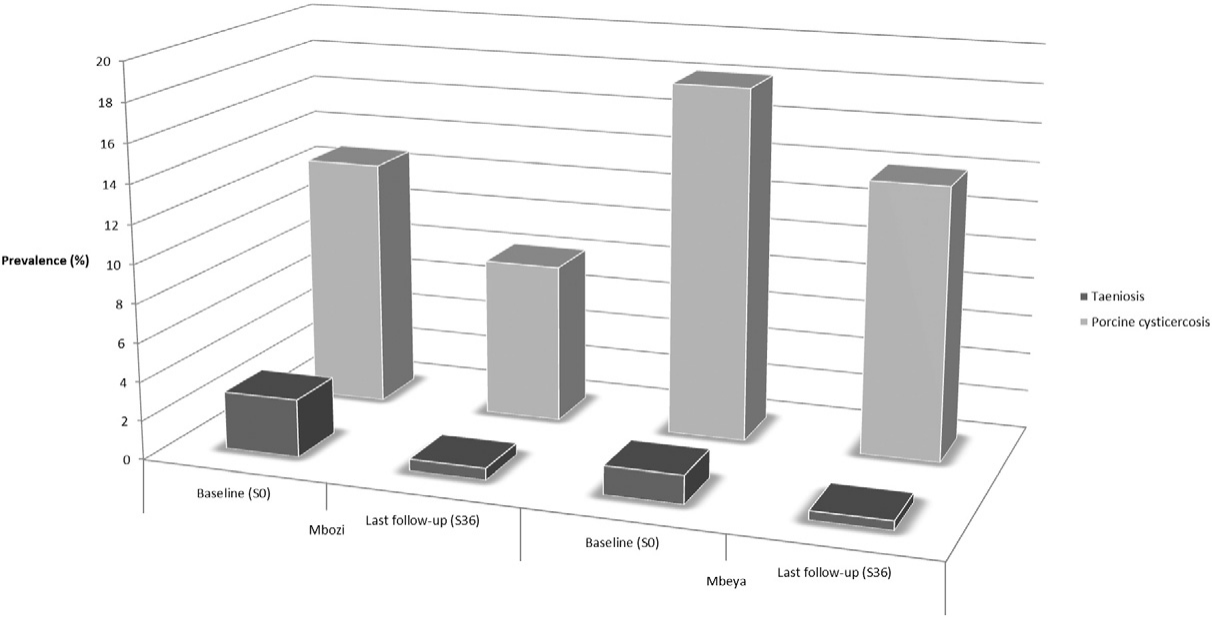
Illustration of the proportional difference in infection of humans and pigs based on prevalence of taeniosis and porcine cysticercosis in Mbozi and Mbeya districts, Tanzania at baseline carried out in Feb-Apr 2012 (S0) and the last follow-up in Jul-Aug 2015 (S36).

The relationship between taeniosis and porcine cysticercosis is illustrated in Fig. 5. In Mbozi district there was an 80% drop in taeniosis prevalence and a 36% drop in prevalence of porcine cysticercosis from baseline to the last follow-up (S36). In comparison there was a 68% drop in prevalence of taeniosis and a 23% drop in prevalence of porcine cysticercosis in Mbeya. This corresponds to 12 percentage points difference in decrease magnitude of taeniosis prevalence and 13 percentage points difference in the decrease magnitude of porcine cysticercosis when comparing the two districts.

## 4 Discussion

This is the first study to assess the impact of control programmes targeting schistosomiasis on *T. solium* taeniosis/cysticercosis measured using both human and porcine outcome variables. The study indicates that a single approach intervention of multiple rounds of MDA targeting a proportion of the definitive hosts for *T. solium* (school aged children) can spill-over into the pig intermediate host population and the untreated adult population. The data presented here further support previous documentation of the positive effect of MDA and “track-and-treat” among school-aged children (Braae et al., 2015b). However, to which extend the ‘track-and-treat’ contributed to the downward trend is currently not quantifiable.

Three studies have been carried out in Latin America using a single MDA of either praziquantel (10 or 5 mg/kg) (Diaz-Camacho et al., 1991, Sarti et al., 2000) or niclosamide (2 g > 6 years, 1 g < 6 years) (Allan et al., 1997) as the intervention with the outcome measured both on human and porcine variables. Allan et al. (1997) carried out “track-and-treat” in two communities followed by MDA. The study revealed a drop in both taeniosis based on copro-Ag-ELISA and in porcine cysticercosis based on enzyme-linked immuneelectrotransfer blot (EITB). However, the study lacked comparison with a control group and options for assessing different treatment strategies. Sarti et al. (2000) showed a reduction in porcine cysticercosis prevalence based on EITB after treatment, but saw no significant change in taeniosis prevalence based on copro-Ag-ELISA. The study also lacked a control group or comparison between interventions. Diaz-Camacho et al. (1991) reported a decrease in both taeniosis and porcine cysticercosis prevalence, but unfortunately this study was based on a small sample size and lacked a control group. Our study is the first from Africa to assess the effect of an existing national control programme on both taeniosis and porcine cysticercosis and the first to show a significant effect in both the human and porcine host populations when comparing two treatment groups.

The association with taeniosis and decreasing age among the school-children in Mbeya could be caused by the younger children who would not have received as many treatments as the older children. Whereas in Mbozi where no association could be seen, children were more likely to have received several treatments regardless of their age, since the MDA was carried out annually compared to biennially in Mbeya. Within the adult population increasing age was a risk factor in both Mbozi and Mbeya, potentially due to increased consumption of pork. To move forward to more comprehensive control of *T. solium* and ultimately towards elimination, assessment of the impact of targeting the adult population in either MDA or a more comprehensive “track-and-treat” is needed.

The prevalence of porcine cysticercosis was consistently lower in Mbozi district compared to Mbeya district despite having significantly less pigs confined at the time. This may indicate that other risk factors are important such as pig management leading to e.g. pigs consuming contaminated feed (Braae et al., 2015a) or water (Komba et al., 2013, Mwanjali et al., 2013).

This study highlights the potential role of MDA as an important tool to control *T. solium*. However, to reach the WHO goals of control of *T. solium* in a foreseeable future (WHO, 2015), additional interventions targeting different stages in the parasite's life cycle will most likely be required (Gabriël et al., 2016, Johansen et al., 2016). Utilising an existing national control strategy targeting schistosomiasis to support the control of *T. solium* is likely to be a highly cost-effective approach. However careful assessment of the populations to be targeted in combination with the implementation of additional intervention tools such as health education and porcine treatment - a One Health approach - is urgently needed to reach the control and elimination goals of these diseases.
